# Recapitulation of the Powassan virus life cycle in cell culture

**DOI:** 10.1128/mbio.03468-23

**Published:** 2024-02-27

**Authors:** Jingwei Song, Jiayao Hong, Chen Yang, Yu Zhang, Zhuoyang Li, Peifeng He, Qiang Ding

**Affiliations:** 1 School of Medicine, Tsinghua University, Beijing, China; 2 SXMU-Tsinghua Collaborative Innovation Center for Frontier Medicine, Tsinghua University, Beijing, China; 3 School of Management, Shanxi Medical University, Taiyuan, China; Virginia Polytechnic Institute and State University, Blacksburg, Virginia, USA

**Keywords:** Powassan virus (POWV), zoonotic disease, encephalitis, replicon, antiviral drug

## Abstract

Powassan virus (POWV) is a tick-borne flavivirus known for causing fatal neuroinvasive diseases in humans. Recently, there has been a noticeable increase in POWV infections, emphasizing the urgency of understanding viral replication, pathogenesis, and developing interventions. Notably, there are no approved vaccines or therapeutics for POWV, and its classification as a biosafety level-3 (BSL-3) agent hampers research. To overcome these obstacles, we developed a replicon system, a self-replicating RNA lacking structural proteins, making it safe to operate in a BSL-2 environment. We constructed a POWV replicon carrying the Gaussia luciferase (Gluc) reporter gene and blasticidin (BSD) selectable marker. Continuous BSD selection led to obtain a stable POWV replicon-carrying Huh7 cell lines. We identified cell culture adaptive mutations G4079A, G4944T and G6256A, resulting in NS2A^R195K^, NS3^G122G^, and NS3^V560M^, enhancing RNA replication. We demonstrated the utility of the POWV replicon system for high-throughput screening (HTS) assay to identify promising antivirals against POWV replication. We further explored the applications of the POWV replicon system, generating single-round infectious particles (SRIPs) by transfecting Huh7-POWV replicon cells with plasmids encoding viral capsid (C), premembrane (prM), and envelope (E) proteins, and revealed the distinct antigenic profiles of POWV with ZIKV. In summary, the POWV replicon and SRIP systems represent crucial platforms for genetic and functional analysis of the POWV life cycle and facilitating the discovery of antiviral drugs.

**IMPORTANCE:**

In light of the recent surge in human infections caused by POWV, a biosafety level-3 (BSL-3) classified virus, there is a pressing need to understand the viral life cycle and the development of effective countermeasures. To address this, we have pioneered the establishment of a POWV RNA replicon system and a replicon-based POWV SRIP system. Importantly, these systems are operable in BSL-2 laboratories, enabling comprehensive investigations into the viral life cycle and facilitating antiviral screening. In summary, these useful tools are poised to advance our understanding of the POWV life cycle and expedite the development of antiviral interventions.

## INTRODUCTION

Powassan virus (POWV) is a tick-borne flavivirus known to induce a severe neuroinvasive disease in humans, occasionally leading to fatalities (1). Two distinct genetic lineages of POWV have been identified: POWV (lineage I) and deer tick virus (lineage II), and these two lineages can only be differentiated through sequencing analysis (2). Recent studies have revealed a concerning trend of increasing human POWV cases in the United States (3, 4). This has raised the possibility that POWV, similar to other pathogens transmitted by Ixodes scapularis ticks, may be considered an emerging human disease in areas where enzootic cycles of POWV, and high populations of I. scapularis ticks coexist (4–6). To date, no prophylactic vaccine or direct antivirals are available to prevent or treat POWV infections (5, 7). Therefore, it is urgent to understand the POWV viral life cycle and develop antiviral strategies.

POWV is an enveloped, positive single-stranded RNA virus, with a genome size of approximately 10,800 nucleotides (2). Upon infection, the viral genome is translated into a single polyprotein, which undergoes subsequent processing by viral and host proteases. This processing results in the generation of three structural proteins, namely capsid (C), premembrane (prM), and envelope (E), along with seven non-structural proteins, known as NS1, NS2A, NS2B, NS3, NS4A, NS4B, and NS5. The structural proteins play a crucial role in virus entry and assembly, facilitating the virus’s ability to enter host cells and form mature viral particles. On the other hand, the non-structural proteins function cooperatively to establish viral replication complexes, essential for efficient viral RNA synthesis (8).

Given the increasing concern over POWV infections, it is imperative to develop experimental systems that contribute to advancing our understanding of the virus and potential therapeutic interventions (9). Flavivirus replicon, a self-replicating sub-genomic system, represents one such valuable system, wherein the genes encoding viral structural proteins are replaced with a reporter gene, such as luciferase or fluorescent protein (10–12). By measuring luminescent or fluorescent signals, the inhibitory effects of compounds on viral RNA replication can be readily assessed. Additionally, this replicon can be stably maintained in cells by incorporating a drug-selectable gene, enabling the streamlining of high-throughput screening (HTS) assays (10, 13). These replicon systems have been successfully established for various viruses, including West Nile virus (WNV) (14), yellow fever virus (YFV) (15), dengue virus (DENV) (10, 16), tick-borne encephalitis virus (TBEV) (17), Japanese encephalitis virus (JEV) (18, 19), and Zika virus (ZIKV) (13, 20), and have significantly contributed to our understanding of viral genome replication in the viral life cycle. However, it is essential to acknowledge that the replicon system alone does not encompass viral entry and virion assembly/release. To address this limitation, flavivirus replicon-based single-round infectious particles (SRIPs) for viruses, such as JEV, DENV, WNV, and TBEV, have been generated by transfection of viral structural proteins into replicon cells (19, 21). The SRIPs are infectious, but progeny viruses cannot be spread from the infected cells as the packaged genome lacks structural protein genes. Thus, the flavivirus replicon system and the replicon-based SRIP system have proven successful in studying the viral life cycle, screening compound libraries for potential antiviral inhibitors, and developing prophylactic vaccines.

In this study, we established the POWV RNA replicon system, demonstrating its application in high-throughput antiviral screening. Furthermore, we established the replicon-based POWV SRIP system, which could recapitulate virus entry, assembly, and release. These systems hold great promise in expanding our understanding of virus–host interaction and accelerating the development of effective treatments for POWV infection.

## RESULTS

### Establishment of a POWV replicon in cell culture

**Fig 1:**
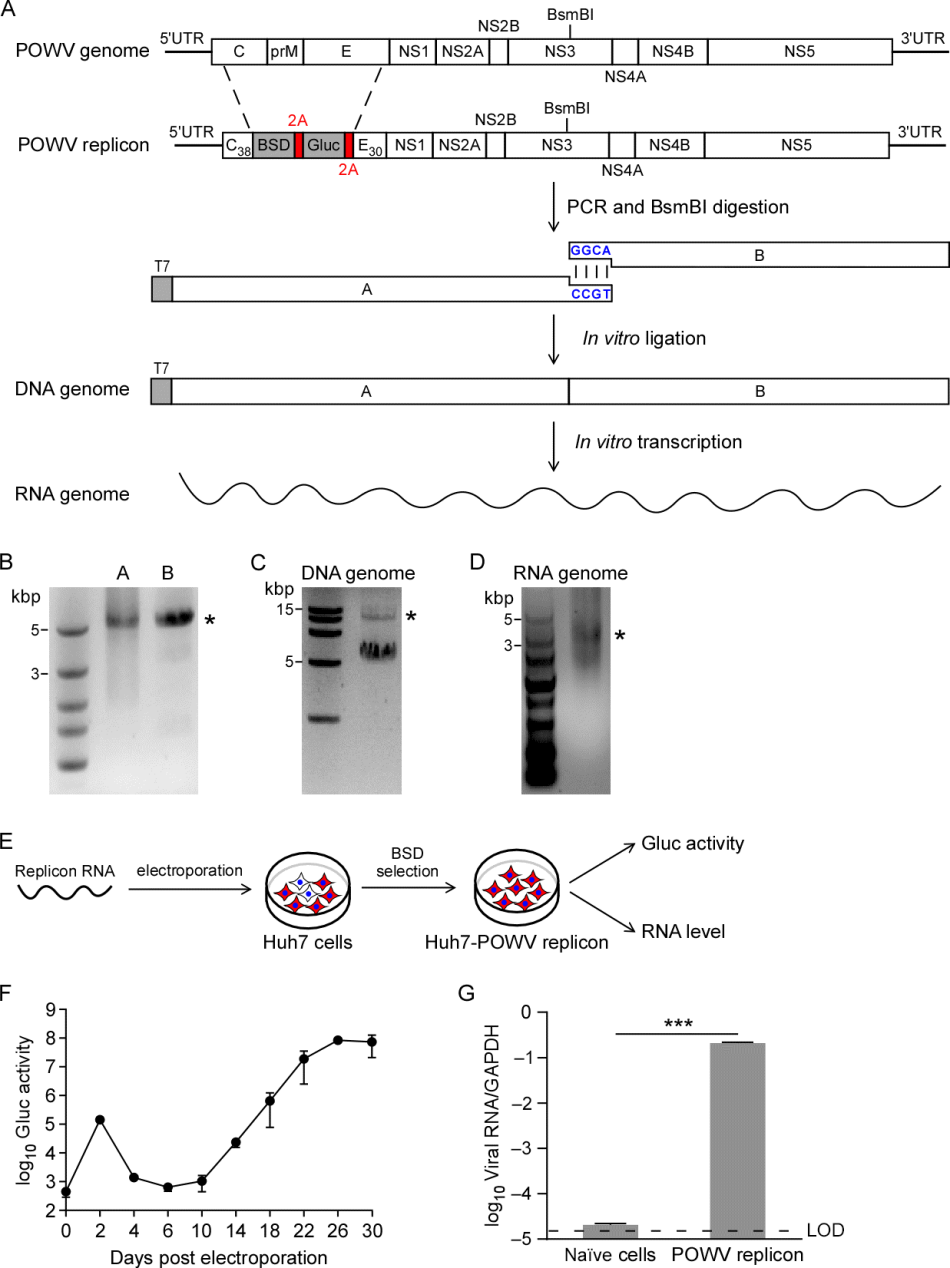
Generation of Powassan virus (POWV) replicon cell line. (**A-D**) Schematic representation of the POWV full-length genome, the POWV replicon genome expressing blasticidin (BSD) and Gaussia luciferase (POWV-BSD2AGluc) and the two fragments used to assemble the replicon genome. The 5′ and 3′ UTRs are indicated with bold black lines; the promoter for the T7 RNA polymerase is indicated with a gray box. The restriction site of BsmBI used for the assembly of the fragments is indicated. BSD, Gluc reporter genes, and Thosea asigna virus-derived autocatalytic peptide 2A (T2A) are indicated with gray and red boxes, respectively. C_38_ represents the first 38 amino acids of the capsid protein, and E_30_ represents the last 30 amino acids of the envelope protein, which serve as signal sequences for the following NS1 protein (panel A). The fragments A and B were amplified using PCR (panel B). The fragment cDNA was digested by BsmBI and purified for directed assembly of POWV-BSD2AGluc cDNA (panel C, and the star indicates the replicon genome-length cDNA), which served as the template for *in vitro* transcription to generate viral RNA genome (see D panel, and the star indicates the replicon genome-length RNA transcript). (**E–G**) The *in vitro* transcribed viral replicon genomic RNA (10 µg) was electroporated into Huh7 cells, and BSD was supplemented into the medium 2 days later. Gaussia luciferase activity were quantified in the cell culture supernatants at the indicated time points (panel F). At 30 days post electroporation, cells were washed and intracellular total RNA was extracted for RT-qPCR assay to quantify POWV RNA. POWV RNA was normalized to the housekeeping gene GAPDH (panel G). The RNA from naïve cells, which were neither transfected with POWV replicon RNA nor treated with BSD, was used as the control. Data represent the mean ± SD (*n* = 3). ***P < 0.001. Significance was assessed by a two-tailed *t*-test. LOD, limit of detection.

We attempted to construct a POWV replicon, which could be used for understanding of virus–host interaction and the discovery of antivirals. To this end, we utilized an *in vitro* ligation approach, which has been used for constructing an infectious clone of SARS-CoV-2 (22–24). We divided the full-length cDNA of the POWV replicon RNA genome, in which the structural genes were replaced with sequences encoding BSD and Gaussia luciferase genes, and a T7 promoter was engineered at the 5′ end of the replicon cDNA for *in vitro* transcription, into two fragments (A and B) based on a type IIS restriction endonuclease site BsmBI, and each fragment can be obtained by PCR using the chemically synthesized viral genome (25) (Lineage I, LB strain, representing the Canadian, Russian, and New York strains) as the template. Specifically, the BSD sequences were positioned downstream of the N-terminal 38 amino acids of the C protein (C_38_). Gaussia luciferase (Gluc) was cloned downstream of the BSD sequence to provide a marker of POWV replication. To ensure correct polyprotein processing and NS1 translocation to the ER, the 2A peptide from Thosea asigna virus (T2A) was incorporated downstream of the BSD sequences and Gluc sequences prior to the C-terminal 30 amino acids of the E protein (E_30_) (Fig. 1A). The two PCR-amplified DNA fragments A and B were digested with BsmBI to generate specific sticky ends (Fig. 1B). The digested fragments were further purified and ligated by T4 DNA ligase to generate the full-length cDNA of the POWV-BSD2AGluc genome, with 9.8 kbp in length (Fig. 1C). Next, these *in vitro* ligation products were used as the template for *in vitro* transcription with the T7 RNA polymerase to synthesize the POWV–BSD2AGluc replicon RNA (Fig. 1D).

To test whether POWV–BSD2AGluc could replicate itself in the cell, *in vitro*-transcribed POWV–BSD2AGluc replicon RNA was electroporated into Huh7 cells to examine reporter gene expression and viral replicon RNA abundance (Fig. 1E F, and G). Upon electroporation into Huh7 cells, the Gluc activity was monitored at an indicated time point, and the BSD (5 µg/mL) was supplemented after 2 days of electroporation. The Gluc signal reached its peak at 2 days post-electroporation and then decreased into background level in the following days, and then the Gluc signal started to increase at 10 days post-electroporation. The Gluc signal increased by 1,000-fold above that of 2 days post-electroporation at day 26 and then reach the plateau (Fig. 1F). At 30 days post-electroporation, the cells were collected to analyze replicon RNA using RT-qPCR, and replicon RNA increased by 10,000-fold above the naïve cells (Fig. 1G).

Collectively, these results indicate that the Gluc signal peaked at 2 days post-electroporation, and the increased Gluc signal and replicon RNA after 10 days post-electroporation represent the translation of the input RNA and the translation of the newly synthesized RNA, respectively.

### Adaptive mutations are able to facilitate POWV RNA replication *in vitro*

**Fig 2:**
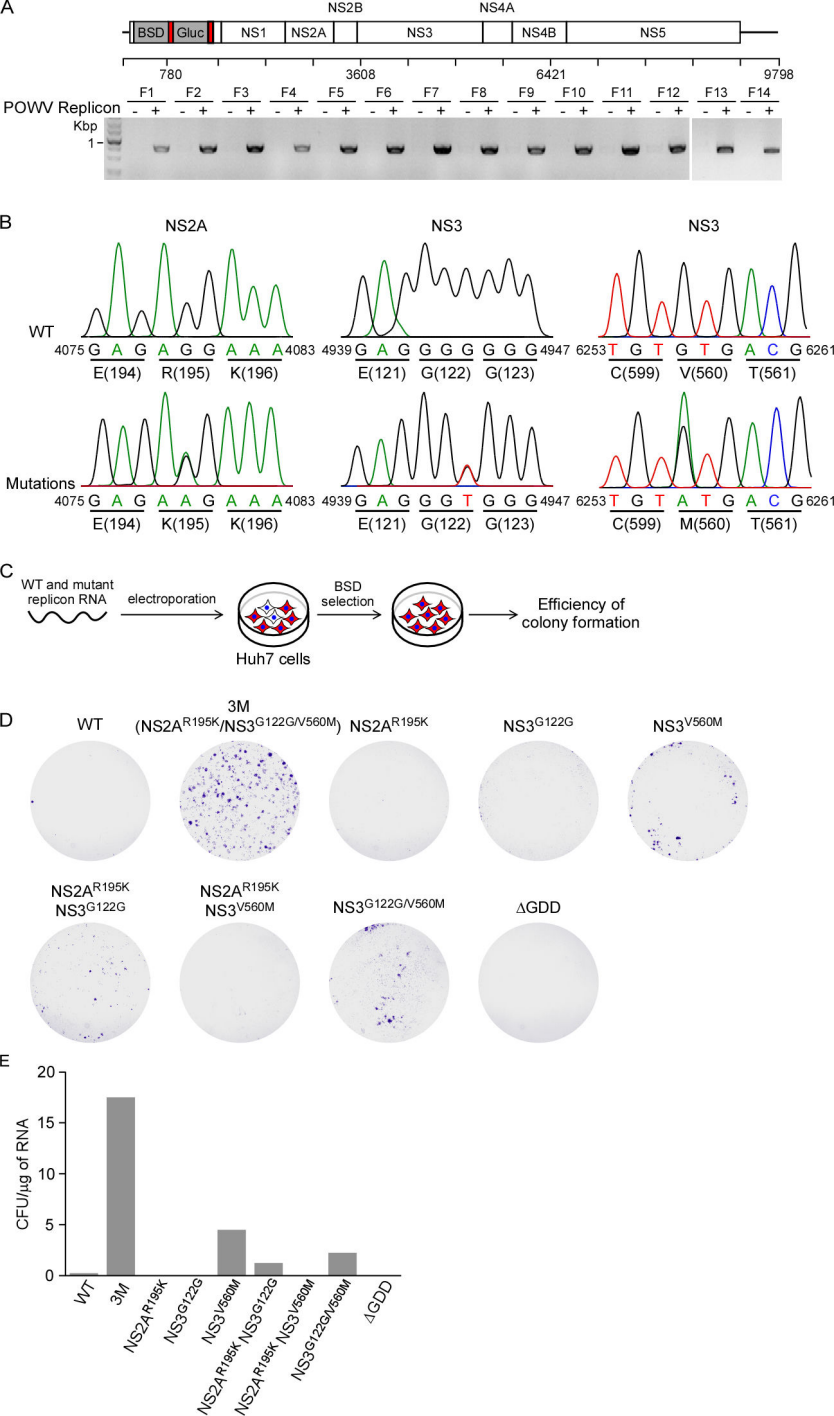
Identification of adaptive mutations in the Powassan virus (POWV) replicon genome. (**A**) RT-PCR was performed with primer pairs to amplify the cDNA across the POWV replicon genome (top panel). The PCR products were resolved on an agarose gel using electrophoresis (low panel). (**B**) Sanger sequence analysis of the PCR products identified the mutations in the viral genomes. The numbers on the schematic refer to the genome of the POWV (Lineage I, LB strain, GenBank: L06436.1). (**C–E**) Huh7 cells were electroporated with 4 µg replicon RNA, and 2 days later, the cells were maintained in the presence of 5 µg/mL BSD for 12 days. Cells were washed, fixed in 4% paraformaldehyde, stained with crystal violet, and counted. As a control, naïve Huh7 cells were electroporated with GDD, an POWV replication defective mutant, and maintained as above in the presence of BSD. Representative data are from three independent experiments.

It is intriguing to note that the POWV replicon exhibits limited replication before 10 days post-electroporation, followed by a marked increase in replication thereafter (Fig. 1F). Given this pattern, we considered the possibility of adaptive mutations emerging in the replicon RNA after 10 days post-electroporation, which could enhance viral replication. To explore this possibility further, we utilized RT-PCR to amplify the cDNA encompassing the entire replicon RNA genome obtained from Huh7–POWV replicon cell pools at 30 days post-electroporation and performed direct sequencing of the PCR products (Fig. 2A; Table S1). These sequence analyses detected three mutations in the replicon genome, G4079A, G4944T, and G6256A, which led to NS2A^R195K^, NS3^G122G^ (synonymous substitution), and NS3^V560M^ (Fig. 2B).

To assess whether these mutations conferred an adaptive phenotype, we introduced the individual or combined mutations into the parental replicon and then transfected the resulting recombinant replicon RNAs (WT, ΔGDD as a negative control with the viral polymerase GDD active site mutated to Ala to eliminate replication activity, individual mutations, double mutations, or triple mutations) into Huh7 cells to evaluate their impact on replicon colony formation (Fig. 2C). Specifically, equal amounts of *in vitro*-transcribed POWV replicon RNA were electroporated into Huh7 cells, and cells were treated with BSD after 2 days of electroporation (Fig. 2C and D). After 12 days of BSD treatment, colonies were observed in WT, 3M (NS2A^R195K^/NS3^G122G/V560M^), NS3^V560M^, NS2A^R195K^/NS3^G122G^, and NS3^G122G/V560M^. Notably, 3M exhibited the greatest effect on the formation of the colonies, with 17.5 cfu/µg RNA, followed by NS3^V560M^ with 4.5 cfu/µg RNA, NS3^G122G/V560M^ with 2.25 cfu/µg RNA, NS2A^R195K^/NS3^G122G^ with 1.25 cfu/µg RNA, and WT with 0.25 cfu/µg RNA. No BSD-resistant Huh7 cell colonies with the replicon were obtained in ΔGDD RNA-transfected cells (Fig. 2D and E). These results suggest that three adaptive mutations synergistically enhance POWV replication.

### Evaluation of the antivirals using the POWV replicon model

**Fig 3:**
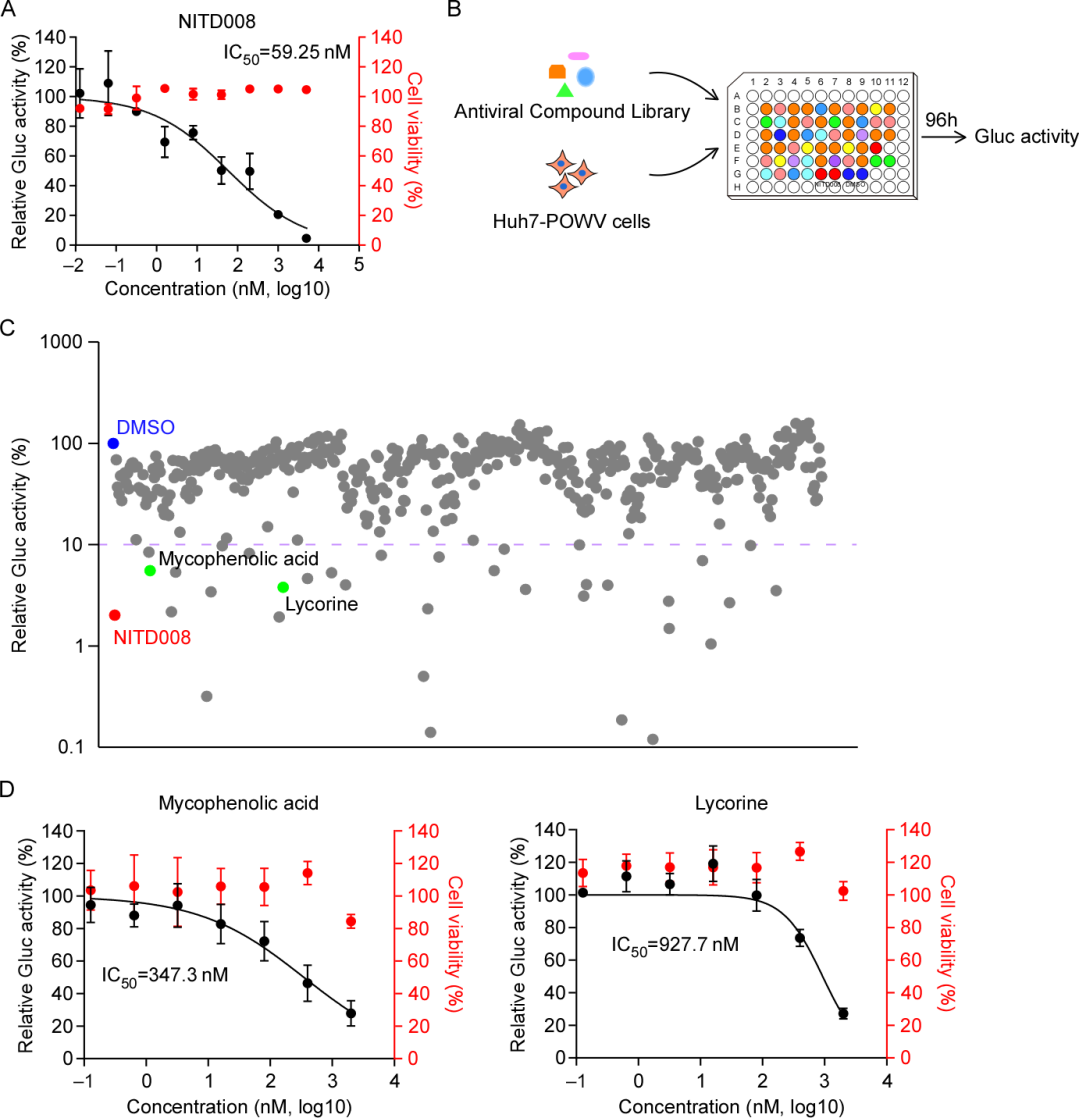
Evaluation of antivirals against Powassan virus (POWV) using a replicon cell culture model. (**A**) The Gaussia luciferase (Gluc) activity in cell culture medium of NITD008-treated cells was quantified at 72 h after treatment. The cytotoxic effect of NITD008 at indicated concentrations was determined using CellTiter-Glo cell viability assay. The virus replication or cytotoxicity is plotted versus compound concentration (*n* = 3 biological replicates). The black dots indicate replicate measurements, and the black lines indicate dose–response curve fits. The red dots indicate cytotoxicity. The IC_50_ values were calculated using Prism software and is representative of one of three independent experiments performed in triplicate. Three independent experiments had similar results. (**B–C**) Screening of 508 compounds from the MCE Antiviral Compound Library and hits selection. The purple dot line represents the threshold (90%) for positive hit compounds. Dimethyl sulfoxide (DMSO) (blue) and NITD008 (red) are used as the control for the screening. Each dot represents a single compound, and the green dots represent the promising candidates, which exhibited potent antiviral activity without a dramatic cytotoxic effect. (**D**) Dose–response curves of selected hit compounds (mycophenolic acid and lycorine). Huh7-POWV cells were treated with different concentrations of mycophenolic acid or lycorine. The IC_50_ values were calculated using Prism software and are representative of one of three independent experiments.

With the growing concern over POWV infections and the absence of antiviral drugs against this virus, the urgency to develop effective antiviral strategies becomes ever more critical (3, 4). Notably, NITD008, an adenosine nucleoside analog, has exhibited promising antiviral activity against various flaviviruses (26–28). Therefore, we aim to assess the *in vitro* antiviral efficacy of NITD008 against POWV replication. For this purpose, we treated Huh7-POWV replicon cells with five-fold serial dilutions of NITD008, commencing at a concentration of 5 µM. After 3 days of treatment, we evaluated the effect of NITD008 on viral replication and cell viability. NITD008 reduced the Gluc signal in a dose-dependent manner, and we calculated the 50% inhibitory concentration (IC_50_) to be 59  nM based on dose–response data (Fig. 3A, left Y-axis); furthermore, no obvious cytotoxicity was observed even at the highest tested concentration of 5  µM (Fig. 3A, right Y-axis). These results not only showed the potent *in vitro* antiviral activity of NITD008 against POWV but also demonstrated the suitability of POWV replicon cell lines for antiviral screening.

In our pursuit to identify additional antiviral candidate compounds against POWV infection, we conducted a HTS of the MCE Antiviral Compound Library, which comprises 508 drugs, using Huh7-POWV cells as the experimental model, and dimethyl sulfoxide (DMSO) or NITD008 were included as the negative or positive control (Fig. 3B). Specifically, 2 × 10^4^ cells were seeded into each well of the 96-well plates, and cell culture medium was collected to assay the Gluc activity as a surrogate of POWV replication. Out of the 508 compounds in the library, we identified 34 hit molecules that exhibited equal or higher inhibition, with an impressive inhibitory efficiency of ≥90%. However, 32 of these hits were excluded due to observable cytotoxicity. We then selected two compounds, mycophenolic acid and lycorine, as the most promising hits for further analysis (Fig. 3C). Next, we performed a dose–response assay to characterize the antiviral activities of these two compounds. Huh7-POWV cells were treated with different concentrations of mycophenolic acid or lycorine. After 72 h of treatment, Gluc was analyzed to reflect virus replication (Fig. 3D). The IC_50_ of each compound was determined; in parallel, the cytotoxic effect of each compound was determined by measuring intracellular ATP levels. As expected, a dose-dependent inhibition of POWV replication was observed with these two compounds (Fig. 3D). The IC_50_ of mycophenolic acid and lycorine were 347.3 nM and 927.7 nM, respectively. Mild cytotoxicity was observed solely at the highest tested concentration of both compounds (Fig. 3D).

Taken together, we developed a stable POWV replicon cell line that could be used as an efficient tool for antiviral evaluation and high-throughput antiviral screening against POWV.

### Establishment of a POWV replicon-based SRIP system to assess the cross-neutralization of ZIKV and POWV

**Fig 4:**
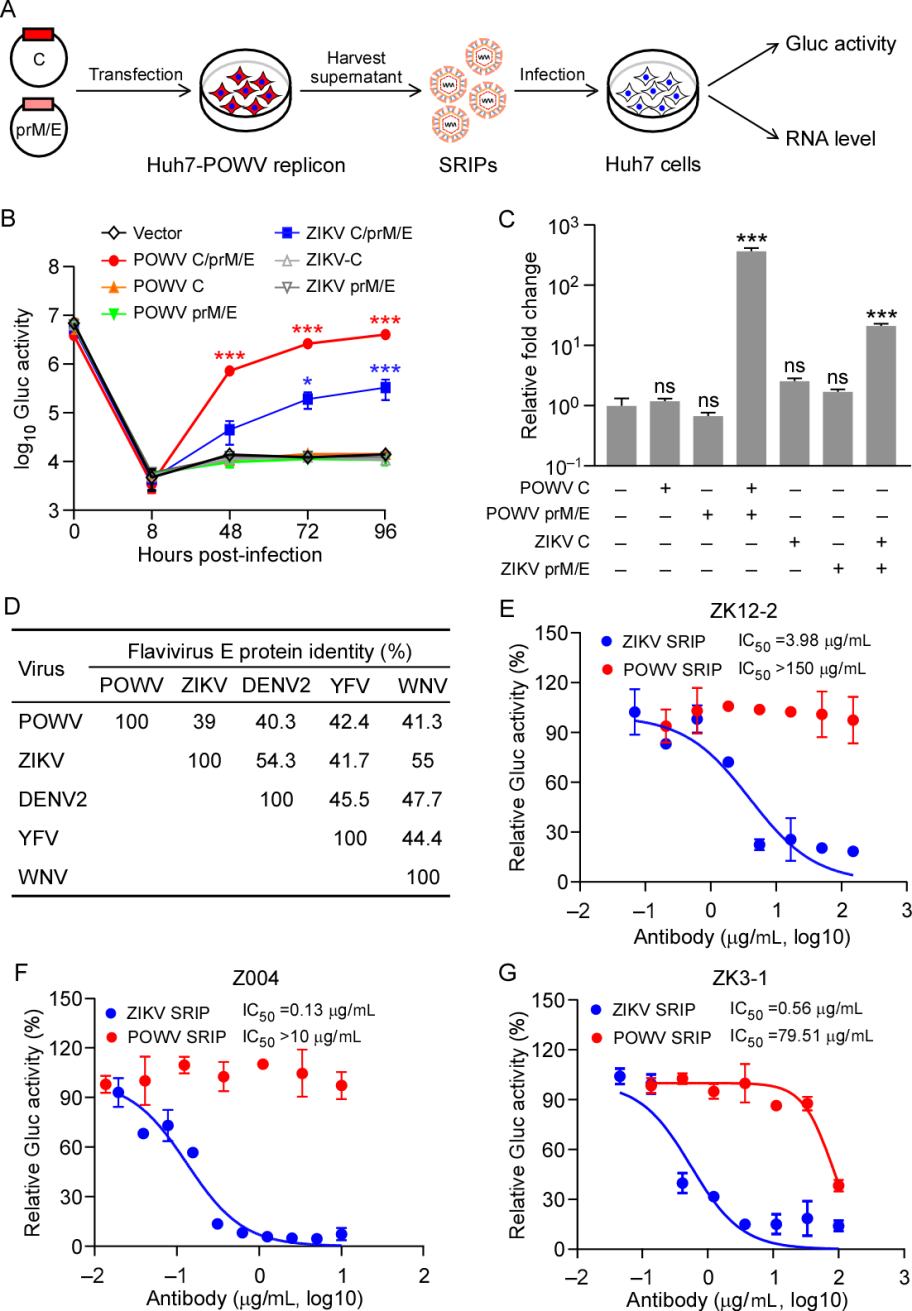
Establishment of Powassan virus (POWV) replicon-based single-round infectious particle (SRIP) system. (**A–C**)A schematic presentation of the experimental procedure used for the production of SRIPs (panel A). The plasmids encoding POWV or ZIKV C or/and prM/E were transfected into Huh7-POWV replicon cells. Three days later, the cell culture medium was collected to inoculate naïve Huh7 cells. Then the cell culture medium was assayed at the indicated time point to determine the Gluc activity kinetics of SRIP infection (panel B), and the cellular total RNA was extracted at last time point for RT-qPCR assay to determine viral RNA abundance (panel C). Error bars represent the SD of the mean from one representative experiment with three biological replicate samples, and all the experiments were repeated three times with similar results. ns: no significance, *P < 0.05, ***P < 0.001. Significance was assessed using a two-way analysis of variance (ANOVA) (**B**) and one-way ANOVA (**C**). (**D**) Analysis of percentages of sequence identity of flavivirus E protein. (**E–G**) Comparison of neutralizing activities of the mAbs against POWV or ZIKV SRIPs. Neutralizing monoclonal antibodies (ZK12-2, Z004, or ZK3-1) were serially diluted and preincubated with POWV or ZIKV SRIPs packaging POWV–BSD2AGluc replicon RNA, respectively. Huh7 cells were infected with the preincubated samples, and at 72 h post-infection readout through gluc secreted into the supernatant was performed. The % infectivity was normalized to SRIP infection without serum incubations. IC_50_ values were calculated using Prism software and is representative of one of three independent experiments performed in triplicate.

As described above, we established a POWV replicon system that faithfully recapitulates viral genome replication (Fig. 1 and 2), but cannot be used to study viral entry and assembly. To overcome this, we endeavored to generate POWV replicon-based SRIPs by providing viral structural proteins in the replicon cells. To achieve this, we transfected plasmids encoding POWV C or/and prM/E into Huh7-POWV cells. In parallel, we also transfected the plasmids encoding ZIKV C or/and prM/E into replicon cells to produce heterologous POWV replicon-based SRIPs. After 3 days of transfection, the cell culture supernatant was collected and then inoculated with naïve Huh7 cells. Subsequently, we washed the cells intensively 8 h after inoculation, and then assessed Gluc activity at indicated time point and determined viral RNA abundance (96 h after inoculation) to detect the SRIP production and infection (Fig. 4A–C). As shown in Fig. 4B, Gluc activity of the Huh7 cells inoculated with cell culture medium from replicon cells individually transfected with the POWV C or prM/E gene was comparable to that of mock-transfected cells. This suggests that no infectious particles were produced in the supernatant when one of these two plasmids was omitted. In contrast, the Gluc activity of the Huh7 cells inoculated with cell culture medium from replicon cells co-transfected with both POWV C and prM/E exhibited a >100-fold increase, indicating the successful production of infectious particles. Additionally, the production of heterologous SRIPs was achieved through co-transfection of ZIKV C and prM/E genes, although the production of ZIKV SRIPs was less efficient than POWV (Fig. 4B). Consistent with the Gluc activity, viral RNA levels increased by 300-fold for POWV and 20-fold for ZIKV in the Huh7 cells inoculated with SRIPs (Fig. 4C). Collectively, these data suggest that infectious POWV or ZIKV SRIPs can be produced by providing viral structural genes (C, prM, and E) into Huh7-POWV replicon cells in *trans*.

Flavivirus E proteins facilitate membrane fusion between the virus and host cell and are the primary viral protein against which neutralizing antibodies are produced (29, 30). Flavivirus E proteins share approximately 40%–50% amino acid identity (Fig. 4D) and are class II fusion proteins with three distinct domains (DI, DII, and DIII). Due to the conserved structural features of the E protein, antibodies generated in response to infection by one flavivirus may have reactivity against others (29, 31, 32). Previous studies have identified several monoclonal antibodies targeting different epitopes of the E protein, such as Z004 (targeting DIII), ZK3-1 (targeting DI/II), and ZK12-2 (targeting DI/II) cloned from convalescent individuals of ZIKV infection could effectively neutralize ZIKV and DENV infections (32, 33). Given these findings, we investigated whether neutralizing monoclonal antibodies against ZIKV could cross-neutralize POWV infection. To assess this, we employed the neutralizing monoclonal antibodies Z004, ZK3-1, and ZK12-2 (32, 33) and measured their neutralizing activity using POWV replicon-based POWV and ZIKV SRIPs as described above (Fig. 4A through C). As expected, ZK12-2 and Z004 were effective against ZIKV, but they failed to neutralize POWV SRIP infection (Fig. 4E and F); ZK3-1 exhibited neutralizing activity against POWV SRIP, with an IC_50_ of 79.51 µg/mL, which was 142 times higher than that observed for ZIKV SRIP (IC_50_ = 0.56 µg/mL) (Fig. 4G). These findings imply that ZIKV neutralizing antibodies demonstrate restricted cross-neutralization capabilities against POWV, highlighting the notably distinct antigenic profiles of these two viruses, particularly concerning the E protein.

## DISCUSSION

Over the past two decades, reported cases of POWV have been steadily increasing, and endemic areas have been expanding (2, 4, 9, 34). Consequently, it becomes increasingly crucial to understand virus biology and develop effective therapeutic strategies. In this study, we have successfully established stable POWV (lineage I, strain LB) luciferase reporter replicon cell lines, which could recapitulate viral replication (Fig. 1 and 2; Fig. S1). In addition, we also found that the NITD008 could effectively inhibit POWV replication (Fig. 3A) and identified that mycophenolic acid and lycorine exhibited potent antiviral activity against POWV replication (Fig. 3C and D). Furthermore, we developed a POWV replicon-based POWV SRIP model, which could be used for the study of viral assembly, release, and entry (Fig. 4). Therefore, we have developed the cell culture models of POWV to study its biology and evaluate antiviral strategies.

We have noted that three adaptive mutations occurred, and they synergistically enhanced POWV replication activity (Fig. 2). These phenomena have been observed for many other viruses, especially those with high mutation rates, such as HCV (35, 36), ZIKV (37), and Kunjin virus (38), as passages in cell culture for prolonged periods of time can result in the accumulation of mutations that often improve virus replication *in vitro* but frequently lead to attenuation *in vivo*. The underlying molecular mechanisms for the cell culture-derived adaptive mutation-mediated enhancement of POWV replication have not been well understood. It is conceivable that these adaptive mutations could facilitate physical interactions among POWV non-structural proteins (NS) or between NS and host proviral factors. Such interactions may, in turn, enhance the assembly of the viral replication machinery. Alternatively, these adaptive mutations might enhance the virus’s ability to evade antiviral immune responses and establish a more conducive environment for POWV replication. Further study is required to dissect the underlying mechanism(s) for adaptive mutation-enhanced replication and the synergy between mutations in NS2A and those in NS3.

In recent decades, viral replicon systems have been successfully established for a variety of RNA viruses, including hepatitis C virus (HCV) (35, 39), DENV (10, 16), WNV (14), ZIKV (13, 20), hepatitis E virus (HEV) (40, 41), SARS-CoV-2 (42, 43), and others. Especially for highly pathogenic viruses, the replicon system offers a safe and efficient means for screening and assessing antiviral compounds. In our study, we have developed a high-throughput antiviral assay utilizing the POWV replicon system and demonstrated its utility in antiviral drug screening and evaluation. Our preliminary results indicate that NITD008, mycophenolic acid, and lycorine exhibited potent antiviral activity against POWV replication in the POWV replicon system. The potential antiviral effects of these compounds against authentic POWV infection *in vitro* and *in vivo* warrant investigation in upcoming studies. Additionally, we intend to evaluate compounds previously recognized for their inhibitory effects against other flaviviruses (44) for their activity against POWV replication using our replicon system. This endeavor is anticipated to uncover additional antiviral compounds effective against POWV infection.

While the replicon model successfully recapitulates viral translation and RNA synthesis in the viral life cycle, it does not encompass viral entry and virion assembly/release. To address this limitation, we developed a replicon-derived SRIPs system by packaging the replicon RNA with structural proteins provided in *trans* (Fig. 4A through C), serving as a valuable tool in basic virology research and antiviral drug discovery and holding immense potential for potential vaccine candidates against the POWV virus. Using POWV SRIP and ZIKV SRIP, we found that the monoclonal antibodies against the ZIKV E protein cannot effectively neutralize POWV SRIP infection (Fig. 4E through G). The observed differences underscore the distinct antigenic properties of these two viruses, particularly in relation to the E protein. This insight into the varying antigenicity of flaviviruses contributes to our understanding of host immune responses and antibody-mediated viral neutralization, emphasizing the importance of tailored approaches in vaccine development and therapeutic strategies for specific flaviviral infections. In further study, monoclonal antibody discovery and characterization will be crucial to the development of a POWV vaccine in the short term as well as a cross-protective flavivirus vaccine in the long term.

Several mosquito-borne flaviviruses, such as YFV, WNV, or TBEV, are classified as BSL-3 agents, necessitating the use of appropriately equipped laboratories when handling infectious virus samples. To address this requirement, viral replicon systems and SRIPs have emerged as valuable alternatives. These systems have demonstrated their effectiveness in studying the complete viral life cycle and facilitating the screening of compound libraries for potential antiviral inhibitors. These significant advancements bring us closer to the development of effective strategies not only against POWV but also other flaviviruses, thereby contributing substantially to global efforts aimed at combating emerging viral diseases.

In conclusion, we have successfully established a POWV replicon cell culture system that faithfully recapitulates viral replication. By utilizing this replicon system, we are poised to gain valuable insights into the intricate mechanisms of POWV replication, the dynamics of virus–host interactions, and the evaluation of potential antiviral drugs. In addition, we also establish the POWV SRIP system based on the replicon, which could be used to model viral entry and virion assembly/release. These innovative tools will undoubtedly contribute significantly to advancing our knowledge of POWV and pave the way for the development of targeted therapeutic interventions in the future.

## MATERIALS AND METHODS

### Cell cultures

Huh7 (ATCC) were maintained in Dulbecco’s modified Eagle medium (DMEM) (Gibco, NY, USA) supplemented with 10% (vol/vol) fetal bovine serum, 10 mM HEPES, 1 mM sodium pyruvate, 1 × non-essential amino acids, and 50 IU/mL penicillin/streptomycin in a humidified 5% (vol/vol) CO_2_ incubator at 37°C. Cells were tested routinely and found to be free of mycoplasma contamination.

### Plasmids

The cDNA encoding POWV (Lineage I, LB strain, GenBank: L06436.1) replicon-BSD-Gluc was synthesized using BGI. The cDNAs encoding C and prM/E of POWV were synthesized using BGI and cloned into pcDNA3.1 vectors. The cDNA encoding C and prM/E of ZIKV were amplified using PCR from the pFK-ZL1 plasmid and cloned into pcDNA3.1 vectors. All of the constructs were verified by Sanger sequencing.

### Assembly of a POWV-BSD2AGluc cDNA by *in vitro* ligation

Fragment A or B were amplified using PCR assay (primers for fragment A: THU-4592 (5′-GGT AAT ACG ACT CAC TAT AGA GAT TTT CTT GCA CGT GTG T-3′) and THU-4595 (5′-ATG CTG TCG TCA CTC GTC TCG TGC CAA TCA ATT CCA CCT TTC-3′); primers for fragment B: THU-4594 (5′-TGG AAT TGA TTG GCA CGA GAC GAG TGA CGA CAG CAT CAG CTG-3′) and THU-5028 (5′-AGC GGG TGT TTT TCC GAG TCA CAC ACC ATC TCC TT-3′)). PCR fragments were digested with BsmBI restriction enzyme (NEB) to get specific sticky end. Digested fragments were then purified using E.Z.N.A. gel extraction kit (Omega). Fragments A (1 µg) and B (1 µg) were ligated by T4 DNA ligase (NEB) in 100-µL system at 4°C for 24 h. At the end of ligation, we took 5-µL product to check the efficiency of ligation using agarose gel electrophoresis. Full-length assembly cDNA was phenol/chloroform extracted, isopropanol precipitated, and resuspended in nuclease-free water for further use.

### RNA synthesis and transfection

The purified POWV-BSD2AGluc cDNA was used as templates for *in vitro* RNA synthesis using the HiScribe T7 ARCA mRNA Kit (NEB). Synthesized replicon RNA was treated with DNase I, followed by LiCl extraction to purify the replicon RNA. Huh7 cells were transfected by electroporation using the following procedures: Trypsinized Huh7 cells were washed with DPBS and resuspended with Opti-MEM I-reduced serum medium (ThermoFisher) at 25 million cells/mL. RNA (10 µg) was mixed with 400 µL of the cell suspensions, transferred to an electroporation cuvette (4 mm), and pulsed at 270 V and 950 μF with the Gene Pulser Xcell Electroporation Systems (Bio-Rad). Transfected cells were immediately transferred to 10-cm culture dishes (Corning) for further culture. Blasticidin (5 µg/mL) was added to the culture medium at 48 h after transfection, and culture medium supplemented with blasticidin was replaced twice a week. Two weeks after transfection, cells were fixed for crystal violet staining.

### RNA isolation and RT-qPCR

Total intracellular RNA was isolated using the RaPure Total RNA Kit (Magen). To analyze the POWV RNA levels in replicon cells, quantitative RT-PCR was performed. In brief, 1 µg total RNA was reverse transcribed using the ReverTra Ace qPCR RT Kit (TOYOBO, FSQ-101) to produce cDNA with random primers. Reactions of qPCR were carried out using the 2× RealStar Green Power Mixture (Genstar, A311) according to the manufacturer’s instructions. The qPCR primers for viral RNA were as follows—POWV: THU-5193 (5′-CGC CCT CAA CAC CAT CAC AAA C-3′) and THU-5194 (5′-TCA ACT CCG TGC TCC TTC AAC C-3′). The sequences of the qPCR primers for GAPDH were described previously (45). Relative expression levels of the target genes were calculated using the comparative cycle threshold method. All data were normalized relative to GAPDH.

### Detection of adaptive mutations

The cDNAs of the POWV RNA replicon were synthesized from total RNA isolated from replicon RNA-transfected cells by reverse transcription reaction. These cDNAs were subsequently amplified with PrimeSTAR Max DNA Polymerase (Takara). Fourteen separate PCR primer sets were used to amplify the entire POWV replicon genome (Table S1). The sequence of each amplified DNA fragment was determined using Sanger sequencing.

### Production of SRIP

Huh7-POWV replicon cells were grown in a 6-well cell culture plate and cotransfected with two plasmids, 1 µg of capsid-expression (pcDNA3.1-C) plasmid and 1  µg of individual prM/E-expression (pcDNA3.1-prM/E) plasmid, using VigoFect DNA transfection reagent (Vigorous). The cell culture medium was harvested on day 3 after the transfection and filtered through a 0.22-µm syringe filter; this medium was used as the SRIP in neutralization tests using Huh7 cells.

### Neutralization assays

Huh7 cells were seeded at 2 × 10^4^ cells per well in 96-well plates the day before infection. Monoclonal antibodies were serially three-fold diluted in DMEM cell culture medium and then mixed with an equal amount of SRIPs. After 1 h pre-incubation of SRIPs with mAb at 37°C, the mixtures were added to the cell monolayers and incubated for 8 h at 37°C, 5% CO_2_. Following incubation, the inoculum was removed, cells were washed with 1× PBS, and finally DMEM medium was added. Supernatants were collected for Gluc analysis as described above at 72 h post-infection, as indicated. Neutralization of the mAb was determined by the percental reduction of Gluc activity in mAb samples compared to Gluc activity in untreated SRIP samples.

### High-throughput screening assays

All compounds were dissolved in DMSO with 5 µM. The HTS assays were established in a 96-well format. Huh7-POWV cells (20,000 cells, total volume: 100 µL) were seeded per well along with different compounds (5 µM). After 96 h of incubation, the cells culture medium collected to assay the Gluc activity using a Renilla Luciferase Assay System (Promega), and the plate was read using the GloMax Discover System (Promega).

### Statistics analysis

Statistical analysis involved the application of either the *t*-test or analysis of variance (ANOVA), coupled with Tukey’s Honestly Significant Difference test, to assess the significance of differences among distinct group parameters. *P*-values of less than 0.05 were considered statistically significant.

## SUPPLEMENTAL MATERIAL

Table S1 mbio.03468-23-s0001.xlsx Information on the primers.

ASM does not own the copyrights to Supplemental Material that may be linked to, or accessed through, an article. The authors have granted ASM a non-exclusive, world-wide license to publish the Supplemental Material files. Please contact the corresponding author directly for reuse.
